# Reducing misdiagnosis in AI-driven medical diagnostics: a multidimensional framework for technical, ethical, and policy solutions

**DOI:** 10.3389/fmed.2025.1594450

**Published:** 2025-10-31

**Authors:** Yue Li, Xin Yi, Jia Fu, Yujing Yang, ChuJie Duan, Jun Wang

**Affiliations:** ^1^School of Humanities and Social Sciences, Shanxi Medical University, Jinzhong, China; ^2^Department of Ideological and Political Education, Shanxi University of Medicine, Medical Humanities Program, Fenyang, Shanxi Province, China; ^3^School of Management, Shanxi Medical University, Jinzhong, China; ^4^Department of Radiation Therapy, Shanxi Cancer Hospital, Taiyuan, China; ^5^Department of Nursing, Shanxi Medical University, Fenyang, China

**Keywords:** artificial intelligence (AI) diagnostics, misdiagnosis risk, AI policy and regulation, patient safety and trust, ethical responsibility

## Abstract

**Purpose:**

This study aims to systematically identify and address key barriers to misdiagnosis in AI-driven medical diagnostics. The main research question is how technical limitations, ethical concerns, and unclear accountability hinder safe and equitable use of AI in real-world clinical practice, and what integrated solutions can minimize errors and promote trust.

**Methods:**

We conducted a literature review and case analysis across major medical fields, evaluating failure modes such as data pathology, algorithmic bias, and human-AI interaction. Based on these findings, we propose a multidimensional framework combining technical strategies—such as dynamic data auditing and explainability engines—with ethical and policy interventions, including federated learning for bias mitigation and blockchain-based accountability.

**Results:**

Our analysis shows that misdiagnosis often results from data bias, lack of model transparency, and ambiguous responsibility. When applied to published case examples and comparative evaluations from the literature, elements of our framework are associated with improvements in diagnostic accuracy, transparency, and equity. Key recommendations include bias monitoring, real-time interpretability dashboards, and legal frameworks for shared accountability.

**Conclusion:**

A coordinated, multidimensional approach is essential to reduce the risk of misdiagnosis in AI-supported diagnostics. By integrating robust technical controls, clear ethical guidelines, and defined accountability, our framework provides a practical roadmap for responsible, transparent, and equitable AI adoption in healthcare—improving patient safety, clinician trust, and health equity.

## 1 Introduction

The integration of artificial intelligence (AI) into healthcare is transforming diagnostic workflows. Machine-learning models now deliver faster and more accurate image interpretation than traditional methods across oncology, cardiology, and radiology (1, 2). Deep-learning systems such as convolutional neural networks (CNNs) can achieve expert-level performance in controlled settings—for example, melanoma detection AUCs exceeding 0.94 (3)—and they show promise for expanding early cancer diagnosis in resource-limited settings (4). Yet these technical achievements do not translate seamlessly to everyday clinical care. Despite benchmark accuracies as high as 94.5% (5), real-world deployments often reveal performance drops of 15–30% due to population shifts and integration barriers (6).

The adoption of AI in diagnostics introduces systemic risks that current governance frameworks are ill-equipped to manage. The World Health Organization defines misdiagnosis as the failure to accurately identify or communicate a patient’s condition (7). Algorithmic opacity and bias further compound this risk. For instance, underrepresentation of rural populations in training datasets has been linked to a 23% higher false-negative rate for pneumonia detection, while melanoma detection errors are more prevalent among dark-skinned patients due to dataset imbalances (8). Additionally, overfitting and spurious correlations can lead to clinically significant false positives, as observed in breast cancer screening (9). Two factors exacerbate these challenges: (1) the “black-box” nature of many AI models, which limits error traceability and undermines clinician trust (10), and (2) blurred lines of accountability among developers, clinicians, and healthcare institutions. We categorize these issues into three failure modes—data pathology, algorithmic bias, and human–AI interaction—outlined in Table 1, which links technical root causes to their clinical consequences.

**Table 1:** Failure modes and root causes of AI misdiagnosis: a technical-clinical analysis.

Failure mode	Technical root cause	Clinical manifestation	Empirical evidence
Data pathology	Sampling bias in training data	Under diagnosis in underrepresented subgroups	28% higher FN rates for dark-skinned melanoma cases (13)
Algorithmic bias	Overfitting to spurious correlations	Over diagnosis of benign nodules as malignant	22% FP increase in lung CT analysis (14)
Human-AI interaction	Automation complacency among clinicians	Delayed correction of AI errors	41% slower error identification vs. human-only workflows (15)

Implementing real-time bias monitoring and interpretability dashboards is crucial to mitigating these issues, but the feasibility and infrastructure requirements must be carefully considered. While these tools could enhance transparency and trust, their deployment in resource-limited settings may face challenges related to cost, data infrastructure, and technical expertise. For hospitals in low-resource regions, the implementation of such technologies could require significant investments in both hardware and training. Therefore, policy recommendations must account for the scalability of these tools, with phased rollouts and tailored strategies to ensure accessibility and effectiveness across various healthcare settings. As noted by Smith and Fotheringham, current liability frameworks inadequately address this tripartite accountability gap, potentially exacerbating health disparities. In line with this, a 2023 study in JAMA found that AI misdiagnosis rates for minority patients were 31% higher than for majority patients in critical care settings (11, 12).

This study addresses these gaps by presenting an integrated framework to reduce AI-related misdiagnosis in real-world care. The framework couples (i) bias-aware data curation; (ii) a hybrid explainability engine that combines gradient-based saliency (e.g., Grad-CAM, Integrated Gradients) with a structural causal model (SCM), aligns the top-k% salient regions with SCM variables, and runs counterfactual/ablation queries with faithfulness checks (deletion/insertion) to yield concise, clinician-facing rationales; (iii) dynamic data auditing via federated learning, whereby each site computes subgroup-stratified metrics (AUC, sensitivity/specificity, ECE, FPR/FNR) locally and shares privacy-preserving aggregates to monitor drift (PSI, KL) and fairness (ΔFNR), with threshold-based alerts and returned reweighting/sampling quotas to mitigate representation disparities; and (iv) accountability-by-design instruments, including versioned model fact sheets and on-chain hashing of artifacts with pointers to off-chain logs for auditor verification. A schematic overview appears in Supplementary Figure S1 (S1A, hybrid explainability; S1B, blockchain-anchored accountability and data flows; S1C, federated learning–based dynamic auditing). Because the work involves no patient intervention or prospective enrollment, clinical trial registration is not applicable.

## 2 Failure modes and risk analysis in AI-based medical diagnosis

Scope of evidence. This is a narrative synthesis and framework paper based on peer-reviewed studies and case analyses; no primary multi-center trial was performed by the authors. Quantitative values cited (e.g., error gaps) reflect external sources explicitly referenced in the text.

### 2.1 Three interdependent failure modes

AI diagnostic errors can be traced to three interdependent failure modes, each demanding targeted mitigation. First, data pathology—driven by sampling biases—leads to systematic underdiagnosis in minority or underrepresented groups, as seen in elevated false-negative rates among dark-skinned patients (13). Second, algorithmic bias—often caused by overfitting to spurious patterns in training data—results in clinically significant false positives, such as unnecessary treatment for benign findings (14). Third, human-AI interaction issues, such as automation complacency or overreliance, can slow down error detection and correction, as demonstrated by delays in clinical workflows when AI is blindly trusted or ignored (15).

Although advanced models such as Vision Transformers can achieve impressive accuracy—for example, an AUC of 0.97 in retinal disease detection (16)—their lack of interpretability remains a major barrier. Clinicians require 2.3 times longer to audit deep neural network (DNN) decisions compared to traditional rule-based systems (17), and 34% of radiologists report overriding correct AI recommendations due to distrust in opaque outputs (18). This underutilization and propagation of errors highlight a critical paradox: as AI models become more powerful, the risks of misdiagnosis, inequity, and accountability gaps can actually increase if transparency and trust are not systematically addressed.

**Figure 1:**
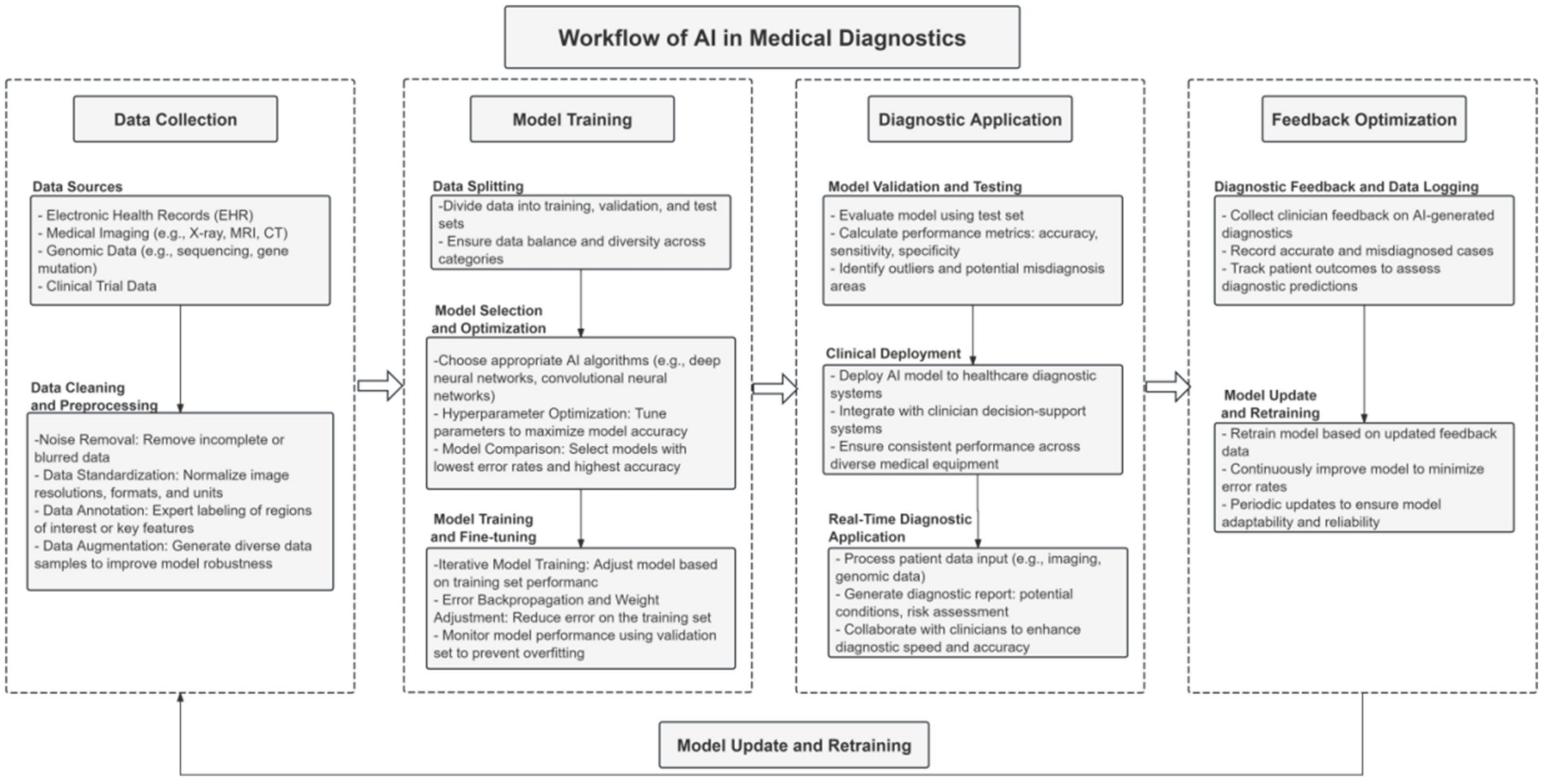
Key workflow of the AI diagnostic system, highlighting critical stages from data collection and model development to clinical deployment and feedback optimization, where technical and ethical vulnerabilities may arise.

As depicted in Figure 1, the end-to-end AI diagnostic workflow—from data collection and model training to clinical application and iterative feedback—includes several points where technical flaws and systemic biases can be introduced and amplified. Each stage represents a potential vulnerability, capable of propagating errors throughout the entire diagnostic process. These interconnected risks underscore the urgent need for solutions that not only enhance technical performance, but also explicitly address the ethical, legal, and operational challenges unique to AI in healthcare.

### 2.2 Data quality, diversity, and accountability in AI diagnostics

The reliability and fairness of AI diagnostics rest on three pillars: data quality and diversity, algorithmic interpretability, and rigorous validation. High-quality, representative data are crucial to avoid systematic disadvantages for minorities. Complex models boost accuracy but may obscure reasoning, limiting clinicians’ ability to verify diagnoses. Rigorous testing, including cross-validation on diverse datasets and real-world clinical trials, is essential to confirm safety and build trust. Table 2 summarizes performance and persistent challenges across key medical fields, providing context for targeted improvements.

**Table 2:** Comparison of AI diagnostic performance across different medical fields.

Diagnostic field	Application	Diagnostic accuracy	Speed	Strengths	Challenges
Dermatology	Skin cancer detection	90–95%	Significantly faster than biopsy	High accuracy for melanoma; valuable for early detection	Struggles with atypical cases and non-Caucasian skin due to data bias (41, 33)
Radiology	Lung cancer detection	85–95%	<1 min per image	Sensitive to small nodules; reduces radiologist workload	Needs high-quality images; susceptible to motion artifacts (14, 42)
Ophthalmology	Diabetic retinopathy screening	90–98%	Immediate (seconds)	Enables mass screening; accurate in staging progression	May miss atypical cases; limited by dataset diversity (43, 44)
Cardiology	ECG interpretation for arrhythmias	85–92%	Real-time analysis	Supports continuous monitoring; aids early detection	Prone to errors in complex or mixed arrhythmias (45)
Pathology	Histopathology for cancer diagnosis	90–97%	Faster than human review	High sensitivity; helps prioritize critical cases	Limited interpretability; risk of over-reliance (46–48)
Pulmonology	Pneumonia Diagnosis via Chest X-Ray	85–93%	Immediate (seconds)	Effective for rapid triage in emergencies	Challenged by overlapping symptoms; sensitive to image quality (49, 50)
Neurology	Stroke Detection on MRI/CT	88–94%	Rapid pre-processing	High accuracy for ischemic/hemorrhagic stroke; time-sensitive	Limited diverse datasets; interpretability issues (51–53)

#### 2.2.1 Data quality and diversity

High-quality, diverse datasets are essential for robust AI performance. If training data are noisy, incomplete, or lack representation from certain racial, age, or geographic groups, models may perform well on some patients but poorly on others, systematically disadvantaging marginalized populations (19–21). For example, suboptimal medical imaging data, including artifacts or poor resolution, can mislead AI systems, leading to diagnostic errors (22, 23). Inadequate data can lead to diagnostic errors, reduce generalizability, and worsen health inequities.

#### 2.2.2 Algorithmic complexity and interpretability

While advanced deep learning models can surpass human experts in detecting subtle clinical patterns, their complexity often comes at the expense of interpretability. Overfitting to spurious details in training data can cause unreliable predictions in new populations (24–27). The “black-box” nature of many models makes it difficult for clinicians to understand, verify, or challenge AI-generated diagnoses, eroding trust and increasing the risk of undetected errors (28, 29). Techniques such as LIME and SHAP improve transparency, but typically offer only partial insights.

#### 2.2.3 Model testing and validation

Thorough external validation, including cross-validation across subgroups and prospective real-world clinical trials, is critical for ensuring AI safety and reliability. Using specialized metrics—such as sensitivity, specificity, and precision-recall—helps confirm performance in clinically relevant terms (30, 31). Following these best practices builds trust among both clinicians and patients.

In summary, progress in these technical domains—data curation, interpretability, and robust validation—is essential to minimize misdiagnosis risk (28). However, technical safeguards alone are not enough. Without clear ethical and legal frameworks, ambiguity in responsibility and accountability can persist, leaving patients vulnerable. The next section addresses these broader challenges, focusing on how responsibility should be allocated and safeguarded in AI-powered healthcare.

## 3 Ethical and legal responsibility allocation in AI diagnostic errors

Technical safeguards alone are insufficient. Ethical and legal responsibility must be clearly defined to protect patients and ensure accountability in AI-assisted medicine. Ensuring responsible and equitable use of AI in diagnostics is not only a technical challenge, but also a profound ethical and legal issue. This section addresses three critical areas: patient safety and equity, accountability gaps among stakeholders, and the evolving standards for patient rights and informed consent.

### 3.1 Patient safety and equity: the ethical stakes of AI misdiagnosis

As AI becomes deeply embedded in clinical diagnostics, misdiagnosis is no longer just a technical failure—it raises fundamental ethical concerns about patient safety and health equity. Diagnostic errors can result in delayed, inappropriate, or unnecessary treatment, directly harming patients. The consequences are often worst for marginalized groups: when AI systems trained on unbalanced datasets underperform for underrepresented populations, existing health disparities are not just maintained—they are made worse (32, 33). Thus, ensuring justice and fairness in AI-supported diagnosis is both an ethical imperative and a technical challenge.

### 3.2 Accountability gaps: roles of developers, institutions, and clinicians

Responsibility for AI errors in healthcare remains ill-defined. Developers are tasked with designing transparent, reliable, and validated systems, yet they rarely interact with patients or clinical realities. Healthcare institutions choose and deploy AI tools, integrate them into clinical workflows, and train staff—but few have established procedures for monitoring, post-market surveillance, or incident response. Clinicians make final care decisions, but may not fully understand or be able to challenge “black-box” model outputs, yet still bear legal and ethical liability. Without clear regulatory frameworks, these overlapping roles lead to confusion, inconsistency, and increased patient safety risks. Practical, shared accountability frameworks tailored to the unique risks of AI-driven medicine are urgently needed.

### 3.3 Patient rights and informed consent in the age of AI

AI-assisted diagnosis introduces new complexities to informed consent. Patients should be told how AI informs their care, its benefits and limitations, and any risks—especially those stemming from model bias or limited explainability. Communicating the workings of opaque models to non-experts is difficult but essential to maintain trust and protect autonomy. In some settings, AI may be the only diagnostic tool available, further reducing patient choice. Ongoing data use by AI systems also raises privacy concerns, making clear, accessible communication about data use and patient rights crucial. Informed consent procedures must be updated to reflect these realities, safeguarding patient interests as AI becomes more prevalent in healthcare.

Practical strategies (≈60–90 s). We adopt a layered, risk-tiered consent approach that fits typical visit time constraints: (i) a one-sentence disclosure (“An AI system will assist your clinician; a human remains responsible for your care.”); (ii) a 30-s “AI Fact Label” in plain language summarizing intended use, key limitations, and any subgroup caveats (e.g., performance may differ in patients >75 years); and (iii) an optional deep-dive explanation accessible via QR/EHR link. Understanding is checked with a brief teach-back (“In your own words, what does the AI add and what are its limits?”). Patients are offered a clear opt-out/human-only review path without penalty. The consent artifact records data use/retention policies and model name/version, and is stored in the EHR. Materials are translated where needed and designed for low health-literacy; in emergencies, deferred consent is documented and completed at the earliest opportunity.

Patient and stakeholder input. To incorporate patient perspectives, we propose a brief, clinic-compatible engagement loop: (i) a 3-item comprehension check after consent (e.g., role of AI, key limits, human-override) and a 5-point trust/clarity rating; (ii) optional focus groups (30–45 min, purposive sampling across age, education, and rurality) to surface concerns and language preferences; and (iii) an auditable EHR record of consent outcomes (accept, opt-out, request human-only review), model/version, and timestamp. Aggregate indicators (e.g., comprehension ≥80%, median trust ≥4/5, opt-out and human-only rates) are reported at the service line and site level to guide content and UI refinements. Materials target ≤8th-grade reading level and are translated as needed. (No new patient data are presented here; future implementations will seek local IRB approval or exemption as appropriate.)

## 4 The role of transparency and explainability in reducing AI misdiagnosis

### 4.1 Why transparency matters

Building on 2.1–2.2—which detail how data pathology and model opacity contribute to diagnostic error—this section focuses on practice-facing safeguards. Transparency is essential for trustworthy AI in medical diagnostics: clinicians who understand how recommendations are generated can validate and act on them more reliably. Providing clear explanations enables secondary review, helping detect hidden errors and improving patient outcomes (19, 24, 25, 34). To avoid the twin pitfalls of undue skepticism and blind trust that can arise with opaque “black-box” models (35), explanations should be concise and point-of-care (e.g., a non-blocking saliency overlay plus a one-sentence causal rationale), paired with explicit statements of system limits and subgroup caveats, and an auditable record of model/version and rationale in the EHR. Such transparency anchors accountability and clarifies when and how AI should be used in practice.

### 4.2 Explainability techniques in practice

Explainability techniques like LIME and SHAP have shown real-world utility in clinical AI workflows. In a retinoblastoma detection study using an InceptionV3 model on balanced cohorts (400 tumorous / 400 normal fundus images), both methods effectively revealed model logic: LIME highlighted tumor regions in individual cases, while SHAP provided feature importance scores across the dataset. This dual insight improved transparency and boosted clinician trust (36).

Similarly, in acute stroke modeling based on random forest or XGBoost, SHAP waterfall plots identified risk contributors such as elevated blood glucose, age, and cerebral blood flow; LIME, meanwhile, localized CT regions that most influenced individual predictions (37). These cases highlight how layered explanations can both guide clinicians and validate AI models.

However, LIME may over-simplify by approximating only locally, and SHAP is often computationally heavy and struggles with feature collinearity—making it less suitable for time-sensitive scenarios (38). Both methods may also miss high-dimensional feature interactions intrinsic to deep neural networks. To address these gaps, we operationalize a hybrid engine that couples gradient-based saliency with an SCM-based causal layer supporting counterfactual queries and ROI ablations; faithfulness and sparsity are monitored to ensure explanations remain clinically actionable (see Supplementary Figure S1A).

Limitations and safeguards. Gradient-based saliency can be sensitive to noise, preprocessing, and ROI thresholds; the SCM layer introduces assumption dependence, and counterfactuals are model-based rather than interventional. We therefore log deletion/insertion faithfulness scores, enforce sparsity, flag saliency–SCM discordance for review, and present explanations as non-blocking overlays to avoid workflow disruption.

Trade-offs and model choice. Where an intrinsically interpretable model (e.g., sparse linear/rule-based or GAM-style) attains performance within a small tolerance of a complex model (e.g., ΔAUC ≤ 0.01–0.02 with comparable calibration/fairness), we prioritize the interpretable model for primary use. When a black-box delivers material performance gains, we retain it with guardrails—pre-deployment faithfulness/stability checks and time budgets, real-time rationale overlays, and prospective monitoring of accuracy, calibration, fairness gaps, and decision latency—while documenting the accuracy–interpretability trade-off in the model’s fact sheet and patient-facing materials.

### 4.3 Patient communication and ethical integration

Transparency in AI is incomplete unless clinicians can translate model reasoning into understandable dialog with patients. This includes clearly explaining AI’s role in the diagnostic process, its capabilities, and its limitations—particularly when performance disparities exist across age groups or demographic segments. For example, saying “This AI system achieves 97% accuracy overall, but it may be less reliable for patients over 75 years old” helps contextualize results, supports informed consent, and reinforces patient autonomy (39). However, explanations must fit clinical workflow constraints. Under pressure, clinicians may lack time to tailor messages; without concise summaries—such as visual markers, standard interpretability labels, or dashboards—technical details risk becoming noise rather than enhancing trust.

Consent-in-practice protocol. At the point of care, clinicians: (1) give the one-sentence disclosure and the AI Fact Label; (2) present a concise rationale from the explainability view (e.g., a saliency overlay plus a one-sentence causal path); (3) perform a teach-back confirmation; and (4) record consent in the EHR, including model/version, date/time, and whether the patient requested human-only review. Explanations are delivered as non-blocking overlays to avoid workflow disruption; language access tools and templated scripts support consistency. In summary, transparency and explainability are not just technical enhancements—they are prerequisites for trust, accountability, and equity in AI-enabled care, and they can be operationalized with brief, standardized communication steps.

Feedback loop and continuous improvement. Patient-reported metrics (comprehension, trust/clarity, perceived usefulness of explanations) and operational signals (time burden, opt-out/human-only rates, teach-back success) are summarized on the communication dashboard and reviewed in monthly huddles with a patient advisory panel. Iterations prioritize brevity and clarity (≤90 s), accessibility (language and format), and equity checks (stratified by age, education, and rurality). Changes to the consent script or UI are versioned and time-stamped to maintain an auditable trail.

## 5 Recommendations and future directions for improving AI diagnostic systems

### 5.1 Technical and ethical strategies to reduce misdiagnosis

Reducing misdiagnosis in AI diagnostics requires both robust technical controls and clear ethical guidelines. First, AI models should be trained on large, diverse datasets that reflect differences in age, ethnicity, and geography, to minimize bias and ensure generalizability. Rigorous validation—using cross-validation, independent test sets, and real-world clinical trials—is critical for uncovering hidden errors and establishing reliability. Furthermore, explainability and transparency must be integrated at every stage of model development. Tools like LIME and SHAP enable clinicians to better understand and trust AI recommendations, making it easier to detect and correct mistakes (40). Combining technical rigor with interpretability is essential for safe and effective clinical use of AI.

#### 5.1.1 Scaling solutions in low-resource settings

Implementing solutions such as blockchain contracts and federated learning audits in diverse healthcare systems, especially those with limited resources, requires careful consideration of feasibility and cost. In low-resource settings, the adoption of these technologies can be challenging due to the required infrastructure, technical expertise, and financial investment. Blockchain-anchored accountability systems, for instance, can introduce costs related to storage, key management, and throughput. We propose a phased implementation approach to scale these tools effectively, starting with pilot projects to assess their viability before broader deployment. By leveraging lightweight blockchain models that store only hashes and timestamps on-chain, we can reduce the data storage requirements, keeping detailed records off-chain and thus minimizing infrastructure costs.

For federated learning audits, which allow healthcare sites to collaborate while preserving data privacy, we recommend starting with local data audits. Each site computes subgroup-stratified metrics and shares privacy-preserving aggregates, which minimizes the need for large-scale computational resources while still enabling essential monitoring functions such as bias detection and data drift monitoring. This approach is particularly suited for resource-constrained settings, where large infrastructure investments are not feasible. We also recommend secure aggregation protocols to mitigate the risks and costs associated with federated learning by minimizing the volume of data transmitted and reducing network overhead. As these audits are scaled, cloud-based solutions could be considered for integrating data from multiple sites without compromising privacy.

#### 5.1.2 Model choice and governance (complexity–interpretability trade-offs)

The preference should be for the simplest adequate model that meets clinical targets, especially in resource-limited settings where computational power and infrastructure are constrained. When an intrinsically interpretable model (e.g., sparse linear/rule-based, GAM-style) performs similarly to a more complex alternative (e.g., ΔAUC ≤ 0.01–0.02 with comparable calibration/fairness), prioritizing the interpretable model helps preserve transparency and reduce resource demands. If a complex, black-box model is necessary for significant performance gains, it is crucial to document the trade-off between accuracy and interpretability in the model fact sheet, specifying clinician-facing explanations and response-time budgets.

Moreover, to ensure that hospitals are ready for deployment, we suggest implementing training programs for clinicians on using blockchain contracts and federated learning systems. Hospitals should focus on educating their clinical staff about the basics of blockchain technology and its use in verifying AI model outputs. Training should include practical demonstrations of how to access blockchain contract logs and use federated learning data audits effectively. This training can be integrated into existing educational programs and can be delivered through workshops or online tutorials. Ensuring that clinicians are familiar with these technologies will promote their adoption and reduce resistance to using these advanced tools in day-to-day workflows.

Prospective monitoring of model performance, including accuracy, calibration, fairness gaps, and decision latency, should be implemented, with human-override options in place if necessary. Periodic reassessment of the model’s performance can help guide decisions about potential simplification to preserve transparency and workflow efficiency. This ensures that the AI system remains effective, interpretable, and scalable in diverse healthcare environments, especially in low-resource settings.

### 5.2 Clarifying responsibility and evolving legal standards

A clear and shared framework for responsibility is urgently needed as AI becomes central to medical diagnostics. Developers must be accountable for model reliability, transparency, and communicating known risks or limitations. Healthcare institutions should evaluate AI tools before deployment, provide clinician training, and monitor ongoing performance, intervening when safety issues arise. Clinicians, while ultimately responsible for patient care, should not be held solely liable for errors that originate from opaque AI models. Regulators must update legal standards and create practical guidelines that distribute accountability fairly and reflect the complexities of AI-assisted medicine.

### 5.3 Advancing ethical standards and policy implementation

Creating a fair and effective AI diagnostic ecosystem requires ongoing collaboration among developers, healthcare providers, policymakers, and ethicists. Ethical standards should mandate fairness, transparency, and respect for patient rights, building on principles such as justice and beneficence. Policies should require data transparency, regular audits for bias, and public disclosure of system limitations. Continuous regulatory oversight is necessary to prevent health disparities and to ensure that technical progress is matched by ethical responsibility. Table 3 provides a consolidated summary of strategic recommendations for enhancing AI diagnostic systems. It outlines technical improvements, ethical considerations, and policy initiatives to guide stakeholders toward a safer, more transparent, and equitable diagnostic framework.

**Table 3:** Summary of strategic recommendations for enhancing AI diagnostic systems.

Category	Issue	Strategy/recommendation	Description
Technical improvements	Data quality & diversity	Data augmentation	Use methods like image rotation, noise addition, and synthetic data to improve diversity.
		Dataset expansion	Include a broad range of demographics, disease types, and medical contexts.
		Data standardization	Standardize labeling and preprocessing to reduce noise and boost accuracy.
	Model complexity	Algorithm optimization	Apply regularization to prevent overfitting and improve generalizability.
		Explainability tools	Integrate SHAP and LIME for better model interpretability.
		Ensemble modeling	Combine multiple models to increase robustness and reduce errors.
	Validation	Cross-validation with diverse data	Validate models on data from different sources and demographics.
		Real-world clinical testing	Deploy models in pilot studies to detect practical limitations early.
Ethical suggestions	Transparency & trust	Data transparency	Disclose data sources, limitations, and processing steps to users.
		Bias monitoring	Regularly check for and correct bias against underrepresented groups.
	Patient consent	Informed consent enhancements	Ensure patients understand AI’s role, limitations, and risks.
	Equity in diagnosis	Inclusive dataset representation	Prioritize diverse data collection to improve fairness.
Policy actions	Responsibility allocation	Accountability framework	Clearly define roles for developers, institutions, and clinicians.
		Guidelines for AI deployment	Set standards for safe AI integration, training, and support.
		Regular audits	Periodically assess AI performance and address bias or risk.
	Patient safety	AI Performance standards	Establish accuracy, sensitivity, and specificity benchmarks.
		Ethics and compliance training	Train staff in AI ethics, safety, and compliance.

Fostering collaboration throughout the AI development lifecycle is crucial for building diagnostic systems that truly serve diverse patient needs. Open-source platforms—such as those pioneered by the Hugging Face community—improve transparency and accountability by making AI models and datasets available for broader review and improvement. Policymakers should also support the adoption of Explainable AI (XAI) frameworks, which make model logic visible and actionable for clinicians and patients alike, directly addressing the “black box” problem and enabling safer, more equitable diagnostic care.

### 5.4 Framework validation roadmap

Validation will proceed in three steps: (i) Feasibility/shadow-mode pilots (1–3 sites) to test non-blocking explainability, bias monitoring, and governance under predefined time budgets; endpoints include calibration (ECE/Brier), discrimination (AUROC), fairness gaps (ΔFNR/ΔAUC), alert precision/recall, and clinician verification time. (ii) Retrospective offline replay with de-identified EHR/imaging streams to stress-test drift detectors (PSI/KL), subgroup metrics, and ledger throughput; report false-alert rate, time-to-detection, and triage effort. (iii) Prospective pragmatic evaluation (cluster A/B or stepped-wedge) comparing standard care versus framework-augmented workflows; primary outcome: misdiagnosis composite; secondary outcomes: decision latency, override rates, calibration/fairness, and patient comprehension. All studies will be pre-registered, include privacy-impact and cost/infrastructure logs, and—where resources are limited—use lightweight deployments (local audits, secure aggregation, hash-only ledger anchoring).

This study has several limitations. First, it presents a conceptual framework supported by a narrative synthesis and secondary sources; it does not include original data collection or prospective clinical trials. Second, reliance on published reports and case descriptions introduces risks of citation and publication bias. Third, the framework’s components—bias-aware curation, hybrid explainability, federated audits, and blockchain-anchored accountability—are not empirically validated here; their performance, costs, and workflow impact may vary across settings. Finally, generalizability is uncertain, especially in low-resource environments with heterogeneous infrastructure and policies. These limitations motivate the validation roadmap outlined below.

## 6 Conclusion

The integration of AI into medical diagnostics holds great promise for improving accuracy, efficiency, and personalized care, but it also introduces risks of misdiagnosis driven by technical limits, model opacity, and diffuse responsibility. This study identifies three core barriers—data bias, lack of transparency, and ambiguous accountability—and advances a coordinated response across technical, ethical, and policy domains. Technically, we call for diverse, representative datasets, rigorous external validation, and explainability that is usable at the point of care (e.g., non-blocking overlays with concise rationales), while explicitly managing the complexity–interpretability trade-off by preferring the simplest adequate model and documenting guardrails when black-box models are used. Ethically, roles are clarified—developers for model quality, institutions for safe deployment and oversight, clinicians for patient care—supported by layered, risk-tiered consent, teach-back, and human-override options. From a policy perspective, we advocate standards that require transparency audits, continuous post-deployment monitoring (calibration, fairness, and decision latency), and context-aware reporting across demographic groups and sites. Aligning these pillars enables stakeholders to harness AI’s benefits while reducing its risks, strengthening patient safety, clinical trust, and health equity.

## Data Availability

The original contributions presented in the study are included in the article/Supplementary material, further inquiries can be directed to the corresponding author.
