# Supplementary material for: Reducing misdiagnosis in AI-driven medical diagnostics: a multidimensional framework for technical, ethical, and policy solutions
Source: Front Med (Lausanne). 2025 Oct 31;12:1594450. doi: 10.3389/fmed.2025.1594450 (PMC12615213; doi:10.3389/fmed.2025.1594450)
Supplement: Supplementary file 1 [file Table_1.docx]

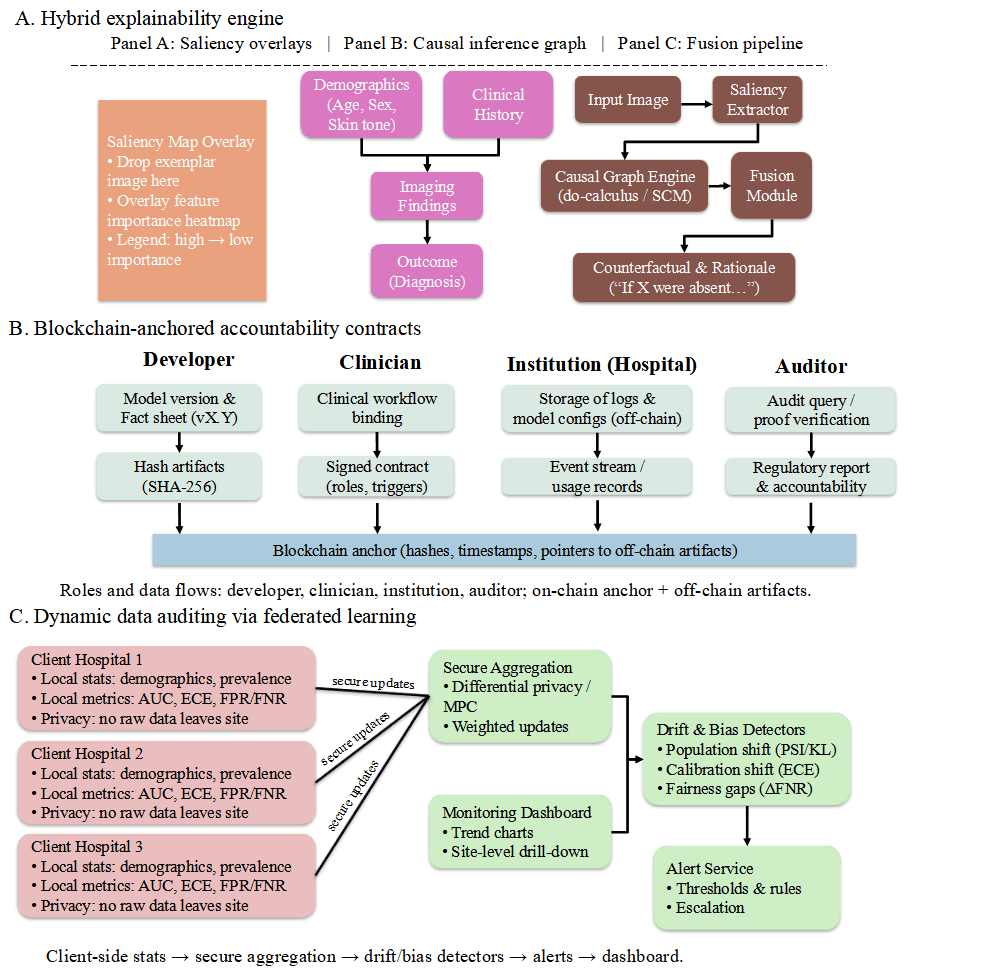


**Figure S1. Integrated framework to reduce AI-related misdiagnosis.**

(A) Hybrid explainability engine. Example image with saliency overlay (left); causal inference graph linking demographics, clinical history, imaging findings, and diagnosis (center); fusion pipeline from Input Image → Saliency Extractor → Causal Graph Engine (SCM/do-calculus) → Fusion Module → Counterfactual & Rationale (right). (B) Blockchain-anchored accountability contracts. Swimlanes for Developer, Clinician, Institution, and Auditor show creation of model fact sheets and contracts, hashing (e.g., SHA-256), on-chain anchoring of hashes/timestamps, and later audit verification that retrieves off-chain artifacts and logs. (C) Dynamic data auditing via federated learning. Multiple client hospitals compute local statistics and performance metrics; privacy-preserving updates are sent to secure aggregation, followed by drift/bias detectors, alerting, and a monitoring dashboard.

Abbreviations: AUC = area under the ROC curve; ECE = expected calibration error; FPR/FNR = false-positive/false-negative rate; PSI = population stability index; KL = Kullback–Leibler divergence; ΔFNR = fairness gap in false-negative rate; MPC = multi-party computation; SCM = structural causal model. Illustrative schematic; no patient-identifiable data are shown

**Methods S1. Litigation-case corpus: data sources and coding framework.**

We performed a structured search of publicly available materials (peer-reviewed case series, malpractice-insurer reports, court/opinion databases, and gray literature) using terms combining AI/algorithm/decision support with diagnostic error/malpractice/litigation (English), covering 2014–2024. Records were de-duplicated across sources.

Inclusion/exclusion. Included records described diagnostic incidents in which an AI/ML system was implicated (assistive, triage, or autonomous) and contained sufficient narrative to code error type and contributing factors. Excluded: non-diagnostic use, editorials without cases, duplicated summaries, or records lacking extractable incident details.

Coding taxonomy. Two independent coders annotated: (i) incident type (missed, delayed, over-diagnosis), (ii) modality/domain (imaging, lab, EHR/triage, other), (iii) AI role (assistive, triage, autonomous), (iv) presence of explainability or rationale in the record, (v) governance artifacts (e.g., audit logs, model versioning, fact sheets), (vi) alleged failure mode (data pathology, algorithmic bias, human–AI interaction), (vii) harm severity (no-harm, temporary, serious, death), and (viii) case disposition (settled, dismissed, plaintiff verdict, defense verdict, unknown).

Reliability & adjudication. After a pilot calibration, coders achieved Cohen’s κ ≥ 0.80 on key fields; disagreements were adjudicated by a third reviewer.

Analysis. We report counts and proportions (Wilson 95% CIs) stratified by modality and failure mode; rates are descriptive and are not interpreted as population incidence.

Ethics & privacy. All materials were public or de-identified secondary sources; no patient-identifiable information was collected.

Limitations. Publicly reported/litigated cases are subject to selection bias; narrative incompleteness may affect coding; categories simplify complex fact patterns..
